# Steel Ball Impact on SiC/AlSi12 Interpenetrated Composite by Peridynamics

**DOI:** 10.3390/ma18020290

**Published:** 2025-01-10

**Authors:** Eligiusz Postek, Tomasz Sadowski, Jajnabalkya Guhathakurta

**Affiliations:** 1 Department of Information and Computational Science, Institute of Fundamental Technological Research, Polish Academy of Sciences, Pawinskiego St. 5B, 02-106 Warsaw, Poland; 2 Group of Solid Mechanics, Faculty of Civil Engineering and Architecture, Lublin University of Technology, Nadbystrzycka St. 40, 20-618 Lublin, Poland; 3 CT-Lab UG (Haftungsbeschränkt), Nobelstr. 15, 70569 Stuttgart, Germany; guhathakurta@ct-lab-stuttgart.de

**Keywords:** interpenetrated composite, impact, damage, peridynamics

## Abstract

Silicon carbide and an aluminum alloy (SiC/AlSi12) composite are obtained during the pressurized casting process of the aluminum alloy into the SiC foam. The foam acts as a high-stiffness skeleton that strengthens the aluminum alloy matrix. The goal of the paper is to describe the behavior of the material, considering its internal structure. The composite’s structure is obtained by using X-ray computing tomography. The thorough computer tomography analysis allows for the high-precision identification of the shape and distribution of the pores in the matrix. The computational model prepared in the framework of the peridynamics method takes into account the pores and their shape. The pores in the structure appeared in the fabrication process. The impact of a steel ball is studied employing the peridynamics method. The sample without any porosity and a porous one were considered during the analyses. It has been found that the porosity of the matrix influences the plastic strain development, but the damage parameter in the skeleton is not affected significantly. The damage advancement in the skeleton during the process is practically identical in both cases. The equivalent plastic strain field is much smoother in a non-porous matrix than in a porous one. The porous matrix has high equivalent plastic strain concentrations, much higher than the non-porous matrix. The shape of the sample is affected by the porosity of the matrix. The sample with a porous matrix tends to fragment, and it shows a tendency towards spallation when in close contact to the surface with the base.

## 1. Introduction

Modern industrial demands constitute the driving force for the elaboration of novel and innovative technologies allowing for manufacturing composites with complex internal microstructure. Various types of composites can be fabricated, consisting of the following:

- Arbitrary ordered or homogenized phase distribution of the materials;
- Directionally oriented layered microstructures;
- One- or multidimensional gradation of the physical and mechanical properties.

Composites are mixtures of different components whose properties are strictly related to the conditions of manufacturing processes. For example, brittle matrix composites and classical ceramic composites are polycrystals made of conglomerates of different grains joined during various fabrication processes, e.g., [1,2,3,4,5]. In the case of ceramic matrix composites, different internal structures were described in detail in [6,7,8,9]. A very complex arbitrarily ordered internal structure has cement matrix materials, e.g., [10,11,12,13,14]. The first problem is to estimate their behavior using various experimental testing methods for the assessment of the physical and mechanical properties of brittle matrix composites [15,16,17,18], including SEM, micro-CT observations, crack propagation at uniaxial or multiaxial mechanical or thermal loadings, 3-point bending, and shear mode of fragmentation. The second problem is the modeling of brittle materials with the application of a micromechanical approach with the analytical model (e.g., [17,18]) or numerical method [19,20,21,22,23,24,25,26].

More complicated internal microstructures have nanoceramic materials and nanocomposite coatings made of nanoparticle powders sintered at high temperatures and pressures [27,28,29].

Another composites class is functionally graded materials (FGMs), e.g., [30,31]. The application of a plastic matrix essentially changes the composite response. Mixing a metallic matrix and ceramic tough grains or other particles leads to forming a metal matrix composite MMC, e.g., [32,33]. A classic example of an MMC is tungsten carbide/cobalt -WC/Co- [34,35,36,37,38] or titanium/molybdenum carbides. More advanced models for estimating the impact behavior, including the thermal effects of the MMCs, were presented in [39,40,41,42].

A fairly new type of novel material are the so-called interpenetrated phase composites (IPCs) consisting of continuous reinforcement in the form of a skeleton (10–20% content), which is filled by a plastic matrix using different technologies, e.g., [43]. The continuity of both phases led to overcoming several performance defects existing in the conventional composites, thus, keeping the virgin properties of both constituent materials. In general, IPCs can be classified as metal/polymer [44,45], metal/metal, and metal/ceramic [46,47,48,49,50] composites. Each type of IPC has a wide range of applications, including aerospace, aviation, automotive, and other construction applications.

In this paper, we will focus on the metal/ceramic IPC Al-Si12/SiC consisting of an aluminum alloy matrix and SiC foam. Kota et al. [48,49] classified various methods of manufacturing IPCs, i.e., ways of filling the ceramic foam with a plastic matrix; the most important are the following:

- Pressureless infiltration, e.g., [51,52];
- Extrusion infiltration;
- Gas pressure infiltration, e.g., [53,54];
- Vacuum infiltration;
- 3D printing, e.g., [55,56].

The mechanical properties of IPCs are related to the volume content of both phases, their spatial distribution, interfacial bonding, and manufacturing methods. The other important parameter for modeling is the interaction between the matrix phase and reinforcement. Due to the complex shape of the 3D ceramic skeleton of IPCs, the pressured infiltration can trigger the destruction of the brittle ceramic reinforcement structure [54,55,56]. However, using open-cell SiC3D foam, which AlSi fills, allows for the creation of two co-continuous internal networks of constituents in the IPC with excellent physical and mechanical properties. This important problem of the interface properties and assessment of the crack propagation was investigated by molecular dynamics for Al/SiC3D in [57,58].

The mechanical behavior of the IPCs strongly depends on the matrix’s porosities spread and the ceramic skeleton’s internal defects. Moreover, the interphase continuity between the metal and ceramic significantly influences the properties of the IPCs, e.g., [59,60]. Their results indicate that the chemical system Al-SiC created at the interface reactive compound Al4C3 leads to a degradation layer. Applying the high silicon content aluminum alloy, e.g., Al-Si12, decreases these effects. However, the interphases are brittle and contain technological cracks after specimen manufacturing [59]. It turned out [61] that adding a small amount of Mg to the aluminum alloy substantially increases the wettability of the interface and finally, its mechanical properties. Additionally, an extension of the pressureless infiltration process for up to 7 h of holding time reduces the porosity of the IPC.

So far, the description of the behavior of the IPCs under dynamic loading is very limited, e.g., [62]. Several papers have been devoted to describing ceramic SiC foam subjected to the crushing process under impact loading, e.g., [63,64]. Very few contributions were related to the assessment of the ballistic properties of IPCs, e.g., [44], where the application of the IPCs led to a reduction in the mechanical impedance mismatch between ceramic plates and metallic ones in various ballistic protection systems.

The reviews presented in [48,65,66] point out that the manufacturing methods of IPCs are still developing. Modifications to obtain a lack of processing-induced flaws and perfect interphases are still required. Furthermore, IPCs with two co-continuous phases are required for an innovative industry. Experimental knowledge of their mechanical, physical, and thermal properties is necessary. In addition, one can observe a lack of advanced numerical models for calculating the equivalent mechanical properties in the literature, including processing-induced flaws under static and dynamic loadings. All of the above data can improve the design of the technological process of IPC fabrication and the methods of their modeling.

This work aims to describe the behavior of the Al-Si12/SiC composite, including its actual internal structure. The elastic–plastic problem in the peridynamics frame is given in Section 2. The composite’s structure is obtained by employing X-ray computing tomography, described in Section 3. The numerical model and the simulation results are presented in Section 4. The formulated numerical model takes into account the shape of the ceramic skeleton and the distribution of voids in the matrix. The impact of a steel ball is studied employing the peridynamics method. For the assessment of porosity influence on the mechanical behavior, two cases were analyzed: the internal structure without porosity and the composite containing voids in the matrix material. It has been found that the porosity of the matrix influences plastic strain development. However, the damage parameter is strongly affected in the skeleton. Further effects, like structure shape during the loading process, spallation, and crack development, are demonstrated.

## 2. Problem Formulation

### 2.1. Elastic Plastic Model with Finite Strains

The material of the matrix is considered elastic plastic with hardening. The plasticity model is Huber–Mises–Hencky [67]. The model is formulated in a finite strains frame. The dynamic equation of equilibrium is of the following form:

(1) $$\rho \ddot{u}\left[x,\;t\right]=\nabla \cdot \sigma^{I}\left[x,t\right]+f\left[x,t\right].$$

**Figure 1:**
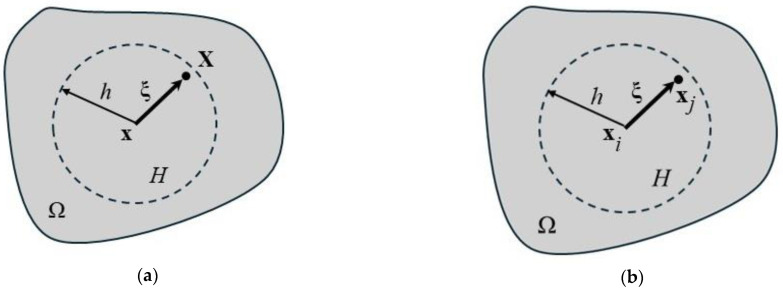
Integration scheme of the equation of equilibrium: (**a**) continuous form; (**b**) discretizetized form.

In the equation above, *ρ* is the mass density, $\ddot{u}$ is the acceleration vector, **x** is the actual position of the body, **f** is the loading vector, and $\sigma^{I}$ is the first Piola–Kirchhof stress tensor. Equation (1) is valid on the domain *Ω* at point x, Figure 1a.

In peridynamics, the equilibrium equation [68] is presented in the form of states [69]. The state-formulated equation of equilibrium in continuous form is as follows:

(2) $$\rho \ddot{u}\left[x,t\right]=\int_{\Omega }\left\{T\left[x,t\right]<x’-x>-T\left[x’,t\right]<x-x’>\right\}d\Omega +f\left[x,t\right],$$

where **T** is the state of force, the brackets < > indicate the vector on which the force-state acts, namely between points **x** and **x**’, and **x**’ and **x**. Point **x**’ lies in in the subdomain *H,* denoted by the circle of radius *h,* later called horizon.

The discrete form of Equation (2) is illustrated in Figure 1b. The summation is performed over the subdomain *H* considering *k* points **x**_j_. The subdomain *H* is of the volume *V*_j_ in the 3D case. Then, the summation is performed over *n* points in the domain Ω. The equation reads as follows:

(3) $$\begin{array}{c} \rho \ddot{u}\left[x_{i},t\right]=\sum_{j=1}^{k}\left\{T\left[x_{i},\;t\right]<x_{j}-x_{i}>-T\left[x_{j},t\right]<x_{i}-x_{j}>\right\}V_{j}+f\left[x_{i},t\right],\\ i=1\;\ldots \;n \end{array}$$

**Figure 2:**
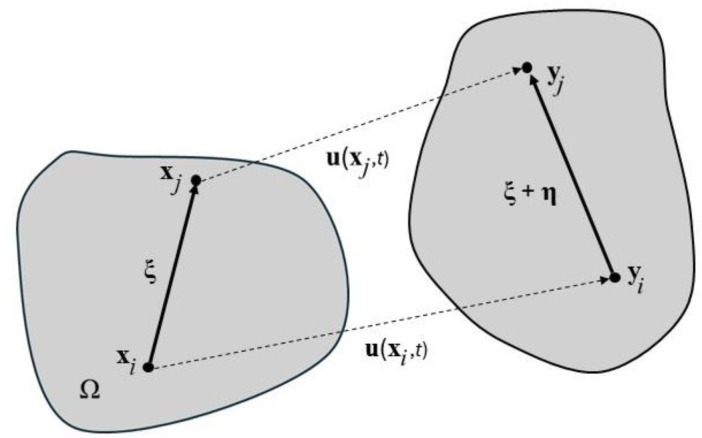
Kinematic relations.

Figure 2 shows the geometrical relations between the undeformed Ω and deformed body Ω’. The vector **ξ** represents the position of the point **x***_j_* with respect to **x***_i_*. The displacement state with respect to the positions of points **x***_i_* and **x***_j_* is as follows:

(4) $$\eta =u\left(x_{j},t\right)-u\left(x_{i},t\right).$$

The deformation state takes the following form:

(5) $$Y\left(x,t\right)<\xi >=y\left(x_{j},t\right)-y\left(x_{i},t\right)=\xi +\eta .$$

The gradient definition reads as follows:

(6) $$F=I+u\nabla_{x}.$$

In finite deformation, the relations between the first Piola–Kirchhof stress tensor **σ**^I^, Cauchy stress tensor ***τ,*** and rotated Cauchy stress tensor ***τ****_rot_* are necessary; namely, they are as follows:

(7) $$\tau_{rot}=R\tau R^{T},$$

(8) $$\sigma^{I}=det\left(F\right)\tau_{rot}F^{-T},$$

where **R** is the rotation matrix obtained from gradient decomposition.

(9) F=RV=VR.

The discrete form of the gradient definition, Equation (6), is expressed as follows:

(10) $$F\left(x_{i},t\right)\approx \left[\sum_{j=1}^{k}\omega \left(\left\lceil \xi \right\rceil \right)\left(Y<\xi >⊗\xi \right)V_{j}\right]K^{-1},$$

where **K** is the shape tensor:

(11) $$K\left(x_{i},t\right)\approx \sum_{j=1}^{k}\omega \left(\left|\xi \right|\right)\left(\xi ⊗\xi \right)V_{j},$$

and **ω** is the influence function [70].

The deviatoric strain rate reads as follows:

(12) $$\dot{e}=d-\frac{1}{3}tr\left(d\right)I,$$

where the deformation rate is the following:

(13) $$d=R^{T}DR,$$

where **D** is obtained from the symmetric decomposition of the spatial velocity gradient:

(14) $$D=\frac{1}{2}\left(L+L^{T}\right),$$

where

(15) $$L=\dot{F}F^{T}.$$

The stress deviator depends on unrotated Cauchy stress tensor **τ**:

(16) $$S=\tau -\frac{1}{3}tr\left(\tau \right)I.$$

The total strain rate is decomposed into elastic and plastic parts:

(17) $$\dot{e}={\dot{e}}^{el}+{\dot{e}}^{pl}.$$

The elastic and plastic parts of the total strain rate are as follows:

(18) $$\;{\dot{e}}^{el}=\frac{\dot{S}}{2\mu },$$

(19) $${\dot{e}}^{pl}=\dot{\lambda }Q,$$

where µ is the shear modulus, $\dot{\lambda }$ is the plastic multiplier, and **Q** is the unit vector normal to the yield surface.

The yield criterion is considered as the Huber–Mises–Hencky (HMH) one:

(20) $${\left(\sigma_{1}-\sigma_{2}\right)}^{2}+{\left(\sigma_{2}-\sigma_{3}\right)}^{2}+{\left(\sigma_{3}-\sigma_{1}\right)}^{2}=2\sigma_{y}^{2}.$$

The yield function is written in terms of principal stress, and *σ*_y_ is the yield strength in uniaxial tension.

### 2.2. Elastic Model with Damage

The case of the state-based model is an elastic brittle model [71,72,73,74]. In this case, a force in a bond is of the following form:

(21) $$f=ce\zeta \left(x,t,\xi \right).$$

In Equation (21), *c* = 18 *k*/(*πh*^4^) where *k* is the bulk modulus, *h* is the horizon, and *e* is the elongation. The force *f* reaches its maximum when *e* = *e_cr_* and drops to 0 when *e > e_cr_.*

(22) $$f=\left\{\begin{array}{c} f_{max}\\ 0 \end{array}\right.\begin{array}{c} \;e=e_{cr}\\ \;e>e_{cr} \end{array}.$$

This is because the function ζ reads as follows:

(23) $$\zeta =\left\{\begin{array}{c} 1\\ 0 \end{array}\right.\;\begin{array}{cc} for & e<e_{cr}\\ for & e\geq e_{cr} \end{array}.$$

The *e_cr_* depends on fracture energy *G_cI_* for mode I, and reads [75] as follows:

(24) $$e_{cr}=\sqrt{\frac{5G_{cI}}{9kh}},$$

where

(25) $$G_{cI}=\frac{\left(1-\nu^{2}\right)K_{I}}{2E}.$$

In Equation (25), ν is the Poisson’s ratio, *K_I_* is the fracture toughness, and *E* is the Young’s modulus.

Finally, the definition of the damage variable reads as follows:

(26) $$d\left(x,t\right)=1-\frac{\int_{\Omega }\varsigma \left(x,t,\xi \right)d\Omega }{\int_{\Omega }d\Omega }.$$

If *d* = 1, the material is fully damaged, namely, the amount of microcracks causes zero load carrying capacity. In case *d* > 0 and *d* < 1, the material is partially cracked. If *d* = 0, the material is sound, and no cracks are there. The integration is performed over *Ω* (Figure 1).

## 3. CT Analysis

**Figure 3:**
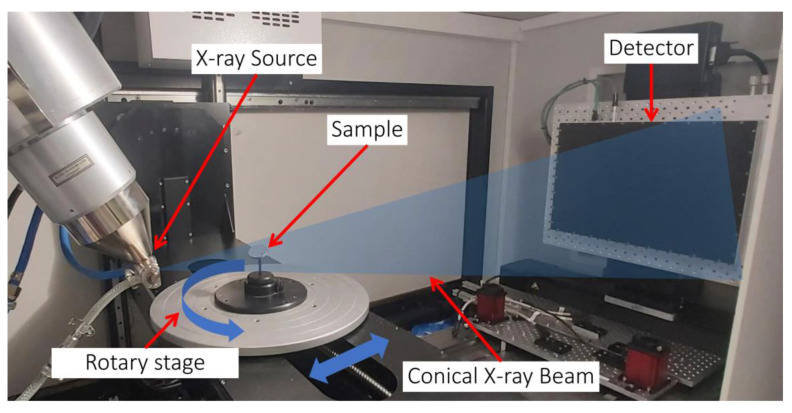
Industrial 3D X-ray computed tomography setup.

Industrial X-ray computed tomography (CT) is a 3D non-destructive measurement technique that captures the outer and inner features of the sample being scanned. Figure 3 shows a CT setup used in the analysis.

The device comprises an X-ray source generating a cone beam on one end and a flat panel X-ray detector on the other. The sample is placed on a high-precision rotary stage between the source and detector, which can move farther or closer to the X-ray source. During the scan, the object is rotated slowly over 360°, and the detector captures several thousand perspectives of the sample.

**Figure 4:**
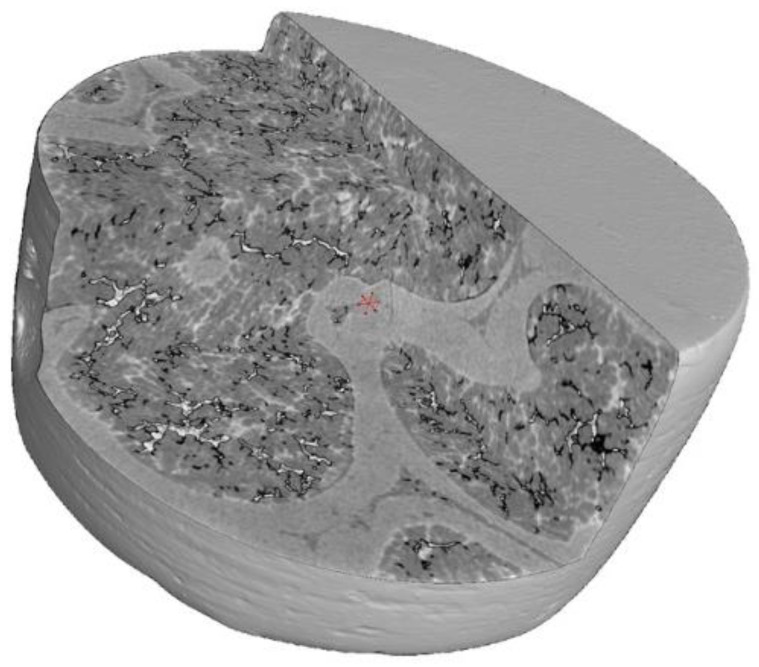
Reconstructed 3D volume.

The 2D images captured by the detectors, known as projections, are then fed into a Feldkamp–Davis–Kress (FDK) reconstruction algorithm to generate a 3D sample volume [76]. If the sample is scanned closer to the X-ray source, the resolution increases, but the field of view decreases. If scanned farther away, the resolution is lower, but a bigger field of view is achieved. The sample size generally limits the position, as the sample must be completely encompassed in the cone beam hitting the detector in all projections. The reconstructed volume is given in Figure 4.

**Figure 5:**
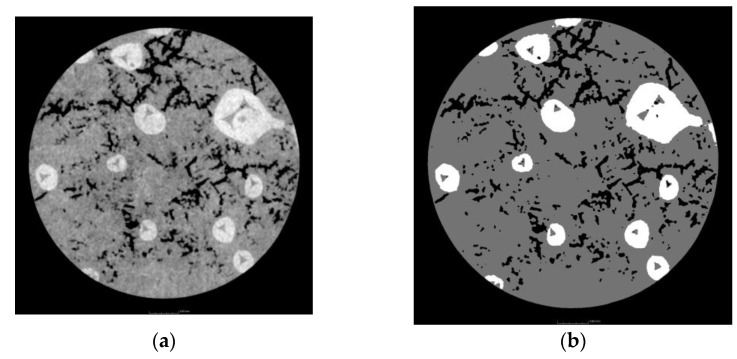
Example of a cross-section, mid-height of the sample: 3D volume filtered with non-local means filter, cross-section (**a**); segmented volume with 2 phases of the composite sample (**b**).

**Figure 6:**
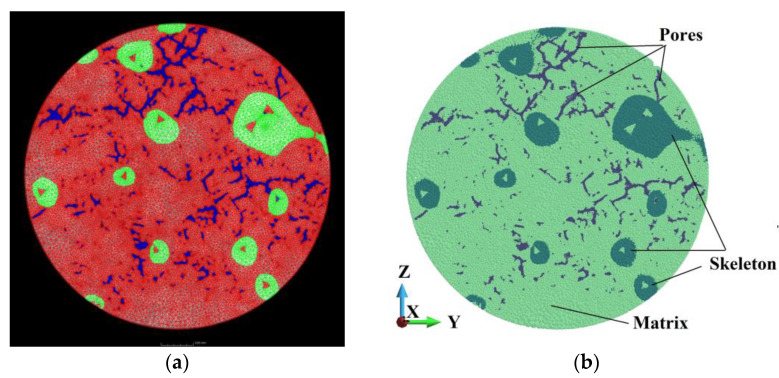
Discretized models: cross-section through a model discretized by tetrahedra (**a**); cross-section discretized by equivalent spheres (**b**).

For the scans presented in this work, the X-ray tube voltage was set to 210 kV and the tube current to 3 µA. A 1 mm Al X-ray filter dampened the soft X-rays and prevented beam hardening artifacts. The detector had a pixel size of 128 µm with 3200 × 2100 pixels. The software processing was performed using VGStudio max 2022.4 [77]. The reconstructed 3D volume was digitally filtered with a non-local means filter to reduce noise. The cross-section at the mid-height of the sample is presented in Figure 5a. This filtered volume is then segmented based on gray value into the two phases of SiC and AlSi2. The cross-section is shown in Figure 5b. After the segmentation, both phases are used to generate a volumetric tetrahedral mesh for the sample; Figure 6a. Then, the tetrahedra are converted into the equivalent volume spheres; Figure 6b. The latter model is suitable for peridynamics.

## 4. Results and Discussion

### 4.1. Numerical Model

**Figure 7:**
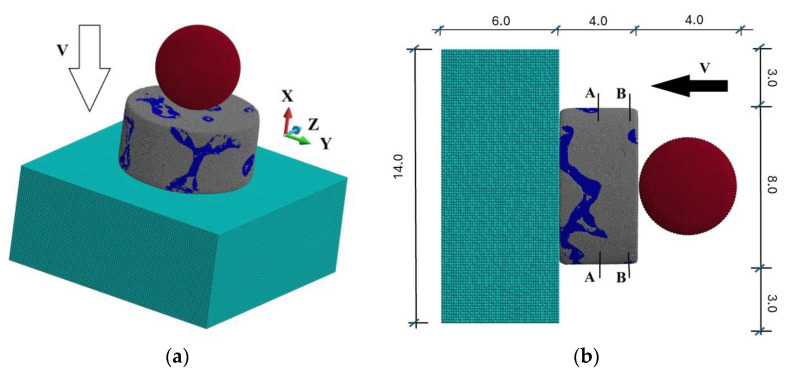
The scheme of the system: (**a**) 3D view of the model; (**b**) side view of the model (the dimensions are [mm]).

The numerical model is prepared for peridynamics analysis. The scheme of the system is given in Figure 7.

The system comprises a steel sphere, composite cylinder, and steel base. The base supports the cylinder, which undergoes an impact of the steel object with velocities of 900 m/s. The cylinder is made of an interpenetrated composite. The composite comprises two phases: silicon carbide (SiC) and an aluminium alloy (AlSi12). In addition, we consider an existence of voids and initial cracks in the matrix. They happen due to an inaccurate filling process.

**Figure 8:**
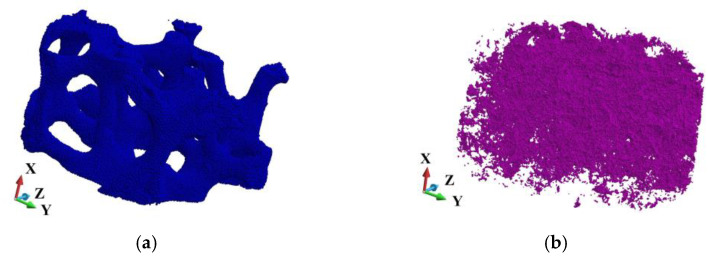
The scheme of the system: (**a**) 3D view of the skeleton; (**b**) view of the cloud of voids.

Figure 8a,b shows the skeleton and the cloud of voids in the sample’s matrix, respectively.

**Figure 9:**
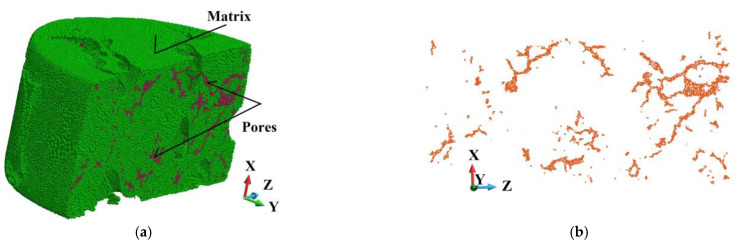
View of the half of the matrix with voids (**a**); pores in the cross-section (**b**).

Since two structure cases are analyzed, two discretizations of the sample are required. In the ideal sample, the matrix counts 4,171,594 volumes and the skeleton 541,273. In the porous sample, the matrix is discretized with 3,570,571 volumes. The skeleton possesses the same number of volumes as in the ideal sample. Both models have the same base and impactor discretizations, namely 500,000 and 277,247 volumes, respectively. The number of volumes arises from applying the tetrahedra discretization of complex shape structures. In particular, the existence of pores, of which the cloud is shown in Figure 8b, forces such a discretization to obtain compatible perfect and imperfect geometries. A system of pores in the matrix is shown in Figure 9a, and the pores are presented in Figure 9b.

The skeleton is made of ceramic SiC material [78]. The material properties of the elastic brittle skeleton are as follows: Young’s modulus is 409.9 GPa, mass density is 3200 kg/m^3^, and Poisson’s ratio is 0.16. Fracture toughness is assumed to be 3.8 MPaxm^1/2^. The e_cr_ is 4.234 × 10^−4^. The elastic–plastic aluminum alloy (AlSi12) matrix has the following material properties: Young’s modulus is 67.0 GPa, mass density is 2700 kg/m^3^, Poisson’s ratio is 0.35, yield limit is 100.0 MPa, and hardening modulus is 1.25 GPa [50]. Based on the derived from CT models, the porosity is 7.9%. The porosity value appeared during the fabrication process of the sample. The base and the impactor are steel with the following elastic properties: Young’s modulus is 210 GPa, mass density is 7850 kg/m^3^, and Poisson’s ratio is 0.3. Aiming to find the influence of porosity, the numerical model undergoes simplifications, assuming the elastic properties of the impactor and the base only.

The simulations are performed using the Peridigm program [70,79]. The explicit time integration technique is applied. The process is followed up to 3 µs with a time step of 1.0 × 10^−5^ µs. The chosen time step is well below the initial stable time step 8.85 ns. The solution is obtained in 30,000 steps. The horizon value is taken as 0.0003 m. A general contact algorithm with a penalty number 1.0 × 10^12^ has been applied. The program is built on a Linux computer cluster with Intel(R) Xeon(R) Platinum 8268 CPU @ 2.90 GHz processors. Each processor has 48 cores and 192 GB memory. The solutions were obtained using 40 processors. The production runs took about 43,000 s, slightly varying due to the current loading of the machine.

The processing of the initial mesh was performed using GiD 16 and MSc Patran 2024 programs [80,81]. The postprocessing and visualization was performed using GiD 16 program.

### 4.2. Damage Analysis of the Skeleton

This section presents a detailed analysis of damage development in the SiC skeleton. It attempts to evaluate the skeleton’s behavior by comparing a fabricated aluminum alloy matrix with the porous one, as shown in Figure 8 and Figure 9.

**Figure 10:**
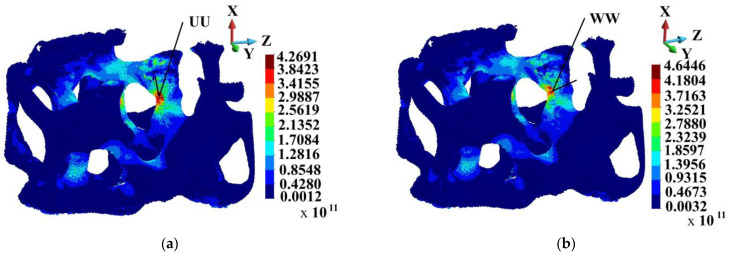
Huber–Mises–Hencky stress distribution in the skeleton; time instance 3.0 µs: (**a**) ideal matrix; (**b**) porous matrix.

In general, porosity in the matrix does not affect the skeleton very strongly. Figure 10 shows the Huber–Mises–Hencky stress distribution in the skeleton. The stress distribution in the skeletons of the ideal and porous matrices are very similar. The maximum HMH stress in the skeletons appears in Points UU and WW, which lie in the same surroundings. The maximum HMH stress is higher in Point WW than in Point UU by 8.1%.

**Figure 11:**
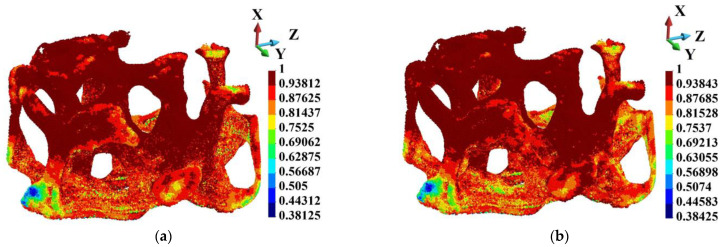
Damage parameter field in the skeleton; time instance 3.0 µs: (**a**) ideal matrix; (**b**) porous matrix.

**Figure 12:**
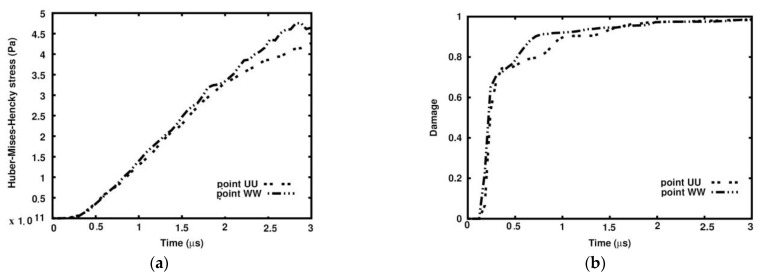
Comparison of Huber–Mises–Hencky stress variability and damage parameter at points UU and WW: (**a**) damage parameter; (**b**) Huber–Mises–Hencky stress.

Figure 11 shows the damage variable field patterns in the skeletons, which are practically the same. In both cases, the skeletons’ structures are almost damaged. The comparison of HMH stress at Points UU and WW shown in Figure 12a indicates that the curves follow the path up to 2.0 µs. Then, the difference starts to become visible, growing to the above-mentioned 8.1% at the end of the process.

Regarding the damage variability at Points UU and WW, it can be noted that the curves grow along the same path steeply up to 0.25 µs. Then, the damage parameter grows slower at Point UU (ideal matrix case) than at Point WW up to 1.0 µs. Further, the curves meet and follow the same path up to 3.0 µs (Figure 12b).

**Figure 13:**
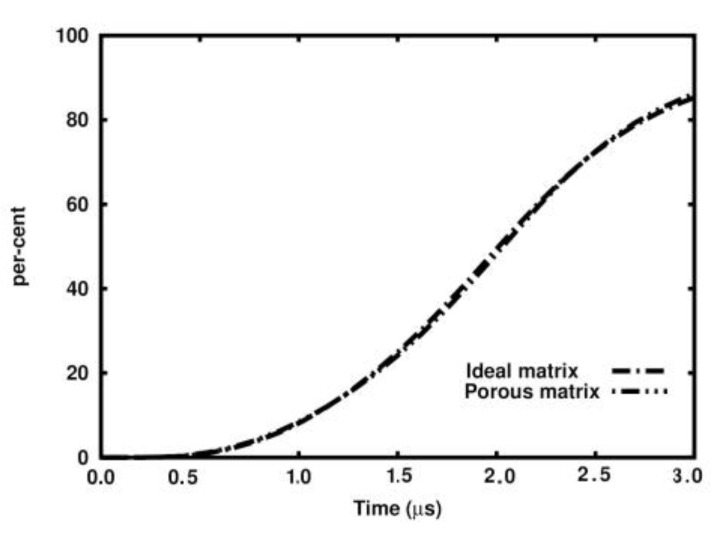
Percentage of damaged volume (d > 80%) of the skeleton in time.

Figure 13 presents the percentage of damaged volume of the skeletons in the ideal and porous samples. The calculation point is considered “damaged” if the damage parameter exceeds 0.8. The curves practically fit each other.

**Figure 14:**
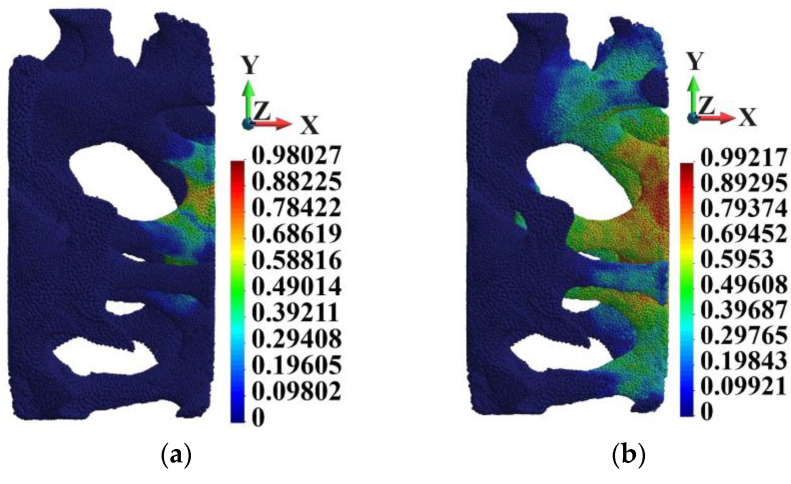

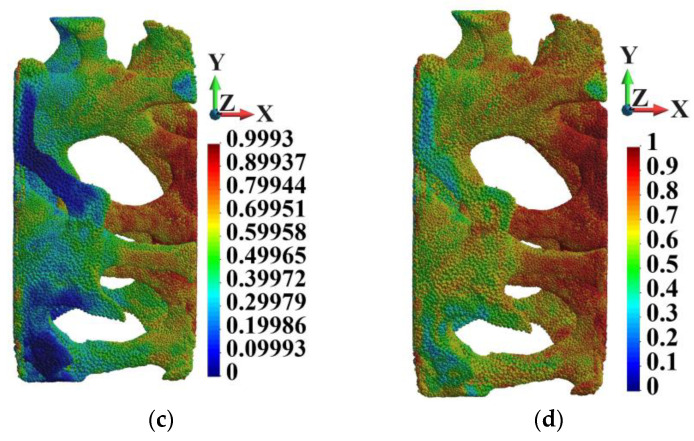
Damage parameter distribution at time instances: (**a**) time 0.24 µs; (**b**) time 0.54 µs; (**c**) time 0.84 µs; (**d**) time 1.14 µs.

Figure 14 shows the damage evolution in the skeleton at four time instances. Due to the similarity of the damage fields in the ideal and porous structures, the skeleton of the ideal matrix has been chosen to present. Four snapshots have been taken.

The damage appears close to the upper surface of the skeleton (Figure 14a) and propagates down towards the lower surface along the branches of the skeleton (Figure 14b,c). The damage follows the stress wave triggered by the impact. The skeleton is practically damaged at time 1.14 µs (Figure 14d). Namely, the damage reached the bottom of the skeleton, and practically, the damage parameter is greater than zero at all points.

**Figure 15:**
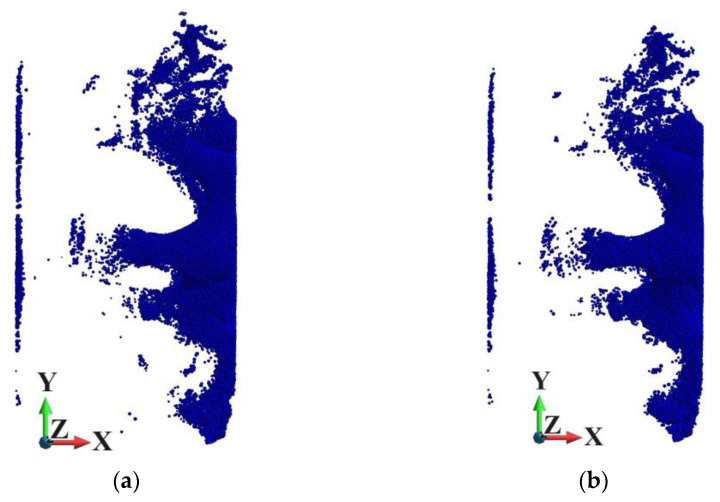
Points where the damage parameter is greater than 08, time 1.14 µs: (**a**) ideal matrix; (**b**) porous matrix.

Figure 15 shows the points where the damage parameter is greater than 0.8 to confirm the similarity of the damage variable fields in both skeletons. The similarity of the distribution of such points in both skeletons is clearly visible.

**Figure 16:**
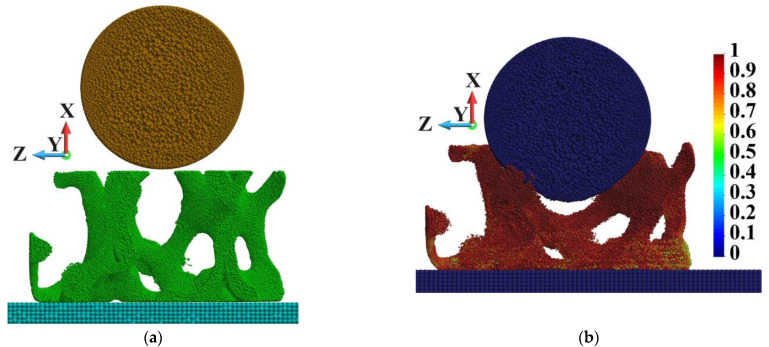
Mid-section in the plane xz: (**a**) initial position of the sphere; (**b**) position of the sphere, shape of the skeleton, and damage distribution at time instance 3.0 µs.

Figure 16 shows the steel ball’s initial (Figure 16a) and final position at the end of the process (Figure 16b). The distribution of the damage parameter is also given there. The entire skeleton is damaged, and the damage parameter is close to 1.0.

### 4.3. Analysis of the Matrix

**Figure 17:**
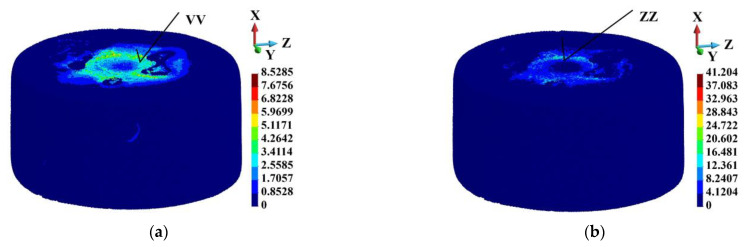
Equivalent plastic strain distribution in the matrix indicating its maximum values (points VV and ZZ), time instance 3.0 µs: (**a**) ideal matrix; (**b**) porous matrix.

**Figure 18:**
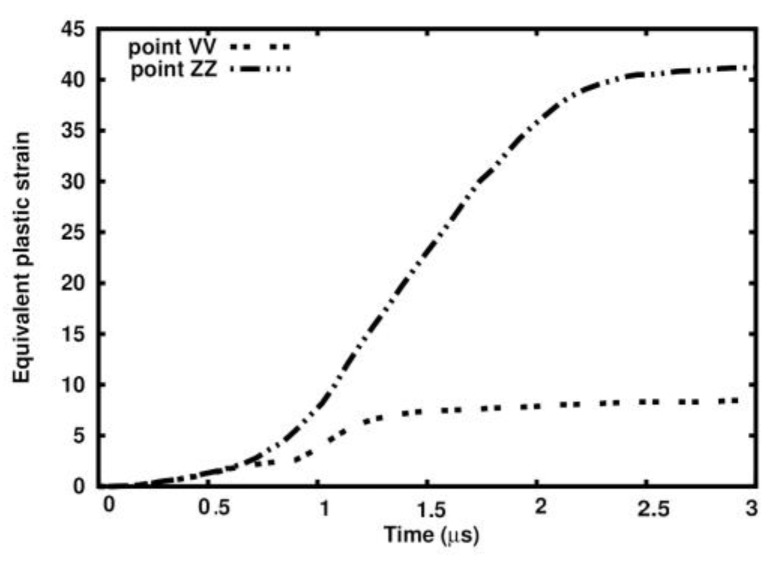
Comparison between equivalent plastic strains at points VV and ZZ in ideal and porous samples.

In the aluminum alloy matrix analysis, the effect of porosity appeared significant. The porosity effect is visible in the equivalent plastic strain distribution. The maximum equivalent plastic strain is concentrated around the sample’s center, where the ball hits the target. The distribution of the equivalent plastic strain is shown in Figure 17. The fields of the equivalent plastic strain are shown in the undeformed configuration for brevity. Points VV and ZZ indicate the places with the highest equivalent plastic strain values. Both points lie close to each other. The dependences of the equivalent plastic strain in time in the spots where they are highest are given in Figure 18. The strain is always higher in the porous sample than in the ideal one. The values are 41.204 and 8.529, respectively, and the ratio is 4.8.

**Figure 19:**
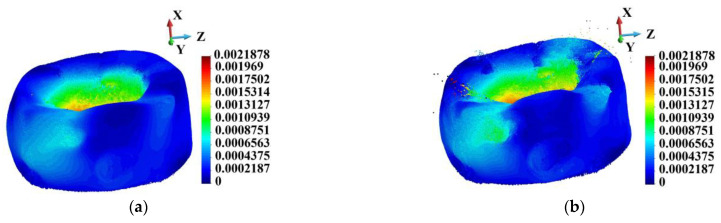
Deformation of the sample and the displacement field distribution, time instance 3.0 µs: (**a**) ideal matrix; (**b**) porous matrix.

Figure 19 shows the samples’ deformed configurations. The displacement range in the porous case (Figure 19b) is set the same as in the ideal case (Figure 19a). The maximum displacement is 0.00414 m in the porous sample. The porous sample is more deformed than the ideal sample. In addition, the fragmented particles are flying off the structure.

**Figure 20:**
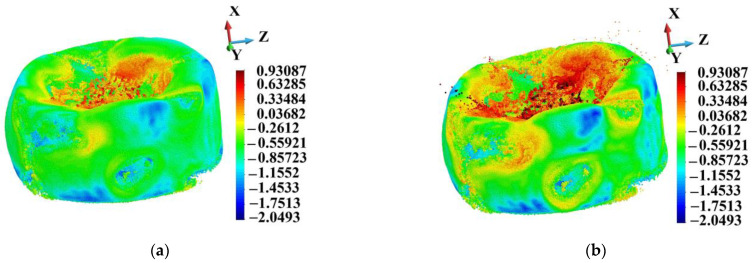
Equivalent plastic strain distribution, logarithmic scale, time instance 3.0 µs: (**a**) ideal matrix; (**b**) porous matrix.

Equivalent plastic strain distribution on deformed samples is presented in Figure 20. The logarithmic scale is used because of high plastic strain gradients. The scale is established following the ideal structure’s minimum and maximum values. It can be noted that the area of the plastic strain of which the decimal logarithm is higher than 0.93087 (8.53) is much larger on the porous sample than on the ideal one.

### 4.4. Analysis of the Cross-Sections

#### 4.4.1. Horizontal Cross-Sections

**Figure 21:**
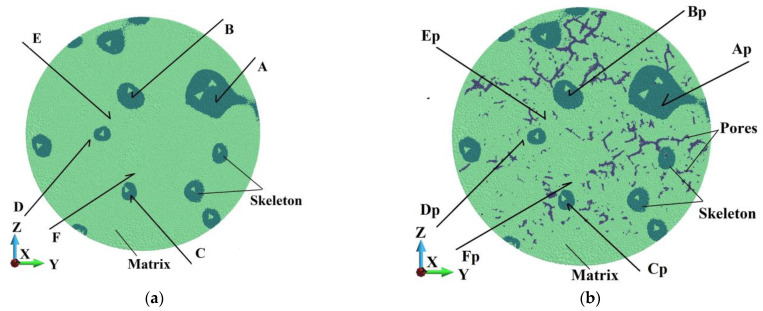
Cross-section (A-A) in the plane yz: (**a**) ideal matrix; (**b**) porous matrix.

**Figure 22:**
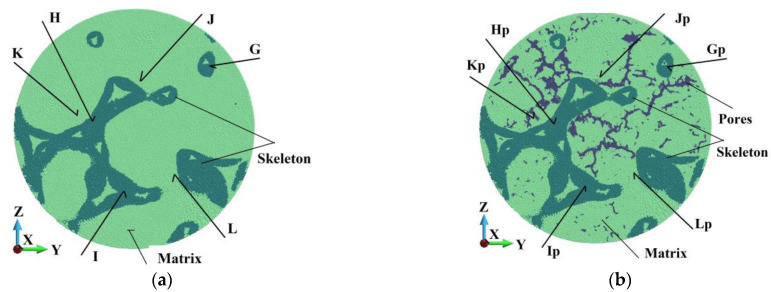
Cross-section (B-B) in the plane xz: (**a**) ideal matrix; (**b**) porous matrix.

Analyzing the samples’ internal structure is necessary to assess and compare their behavior. Following Figure 7b, we consider the horizontal mid-cross-section (A-A) and a cross-section B-B lying close to the upper struck surface. In addition, a vertical cross-section in the middle of the sample in the plane x-z is chosen. A detailed view of the horizontal cross-sections is shown in Figure 21 and Figure 22.

In Figure 21 and Figure 22, selected groups of points have the same position. Namely, Points A, B, and C in the skeleton in Figure 21a correspond to Points Ap, Bp, and Cp in the skeleton, but the sample possesses a porous matrix (Figure 21b). In an analogy, Points G, H, and L in the skeleton in Figure 22a conform to Points Gp, Hp, and Ip in the skeleton in Figure 22b. The same key is used for the points in the matrix. In cross-section A, Points D, E, and F are relevant to Points Dp, Ep, and Fp in Figure 21a,b, respectively. Points in the matrix in Figure 22a,b, namely, J, K, and L, suit Points Jp, Kp, and Lp.

**Figure 23:**
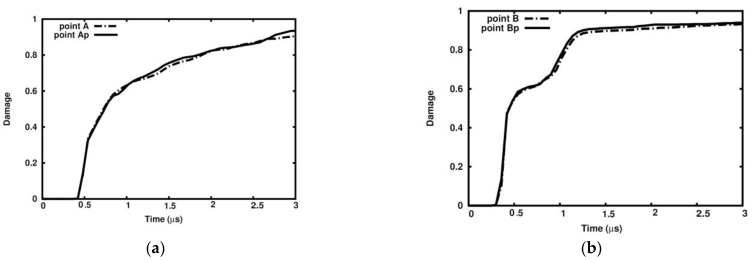
Damage dependence in ideal matrix and porous matrix: (**a**) Points A and Ap; (**b**) Points B and Bp.

**Figure 24:**
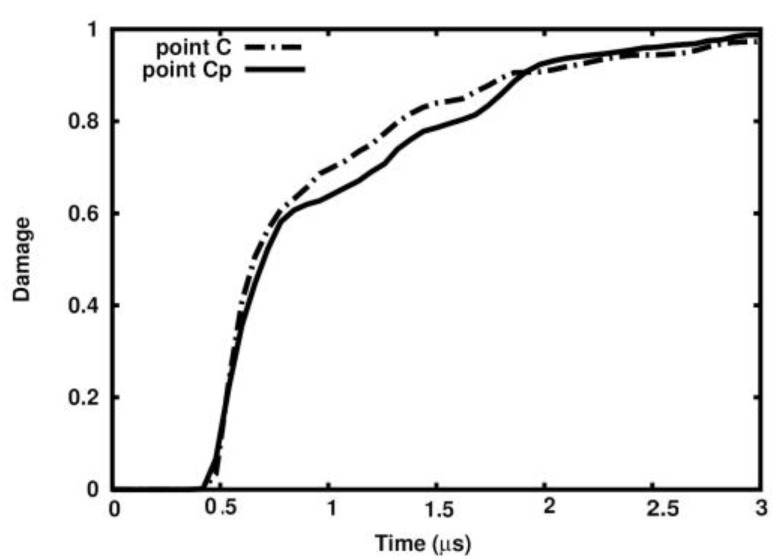
Damage dependence on time in ideal matrix and porous matrix at Points C and Cp.

Figure 23 and Figure 24 show the damage parameter dependency on time in Points A, B, and C in the ideal sample and in the relevant points Ap, Bp, and Cp in the porous one. The pair of curves appear to follow practically the same path, which means that the damage parameter is not affected by the sample’s porosity.

**Figure 25:**
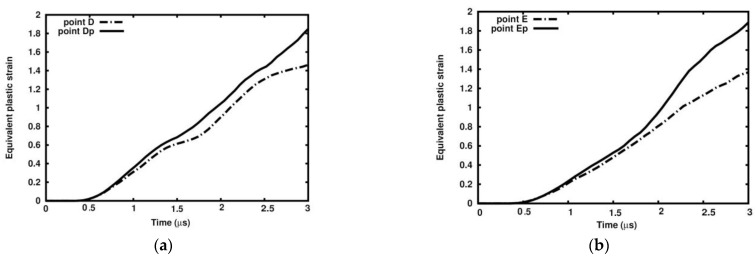
Equivalent plastic strain dependency on time: (**a**) Point D and Dp; (**b**) Point E and Ep.

**Figure 26:**
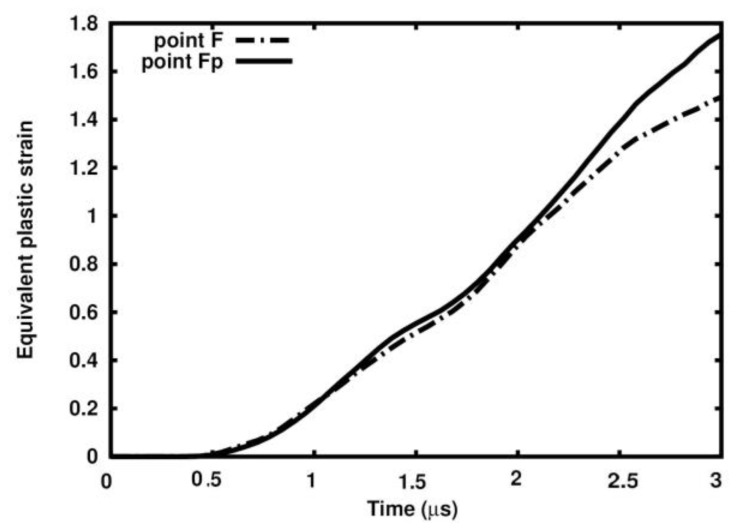
Equivalent plastic strain dependence in ideal matrix and porous matrix at Points F and Fp.

The equivalent plastic strain evolution is followed in Points D, E, and F and Points Dp, Ep, and Fp (Figure 25 and Figure 26). At the end of the observed time interval, the equivalent plastic strain is always more significant in the porous sample than in the ideal one.

The equivalent plastic strain values in Points Dp, Ep, and Fp are 26%, 38%, and 17% higher than in relevant points in the ideal sample.

**Figure 27:**
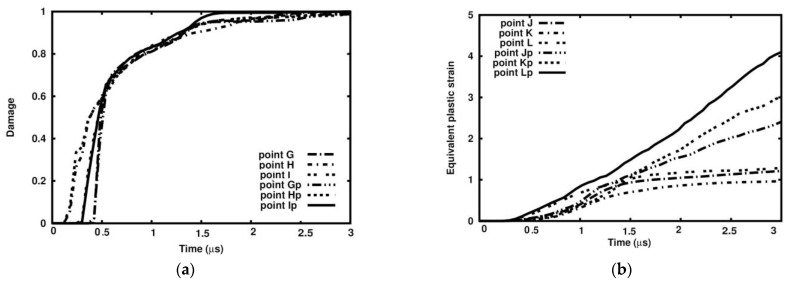
Variable dependency in selected points in the cross-section (B-B) in selected points: (**a**) damage parameter; (**b**) equivalent plastic strain.

In cross-section B-B, the dependence of the damage parameter on time is similar to that in cross-section A-A in the sense that the curves run practically along the same paths (Figure 27a). All curves tend to approach 1.0 within 2.5 µs.

Figure 27b presents a comparison of equivalent plastic strain evolution in Points J, K, and L and in Points Jp, Kp, and Lp. The difference in the values at time 3.0 µs is significant. Namely, it reads 97%, 214%, and 218%.

**Figure 28:**
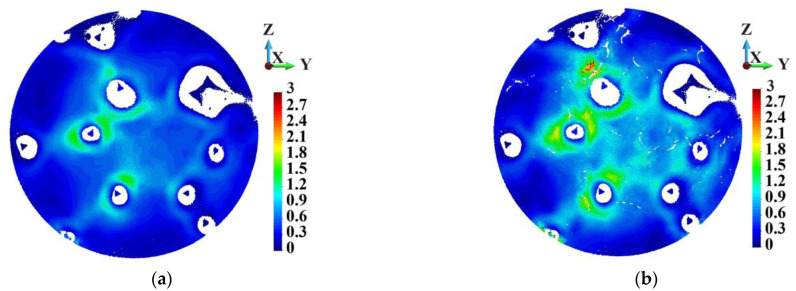
Equivalent plastic strain distribution in the matrix, cross-section A-A: (**a**) ideal; (**b**) porous.

**Figure 29:**
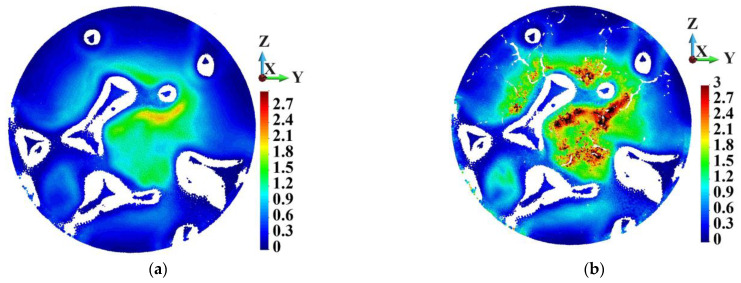
Equivalent plastic strain distribution in the matrix, cross-section B-B: (**a**) ideal; (**b**) porous.

The distribution of the equivalent plastic strain in the cross-sections A-A and B-B is presented in Figure 28 and Figure 29. The colors blue to red denote the values between zero and 3.0. The ranges and scales of colors are set common for both figures. This is done because the higher values are intensely concentrated. Therefore, the equivalent plastic strain higher than 3.0 is marked in black. The most intense plastic strain concentration in cross-section A-A is close to the skeleton in the sample with the ideal matrix (Figure 28a). In the case of cross-section B-B, the plastic region of high plastic strain is focused on the center since the cross-section B-B lies close to the impacted surface (Figure 29b). The highest equivalent plastic strains in the ideal sample in the cross-sections A-A and B-B are 1.644 and 2.14, respectively. In the porous sample, the picture of the equivalent plastic strain differs from the ideal one. Observing Figure 28b and Figure 29b, one notes more extensive areas of the equivalent plastic strain higher than 1.2 (yellow to red colors) than in Figure 27a and Figure 28a. The maximum equivalent plastic strains in the cross-sections A-A and B-B are 6.234 and 9.222, respectively. The values are 3.8 and 4.3 times higher than in the ideal structure. The equivalent plastic strain is the highest close to the pores (Figure 29b).

#### 4.4.2. Vertical Cross-Sections

**Figure 30:**
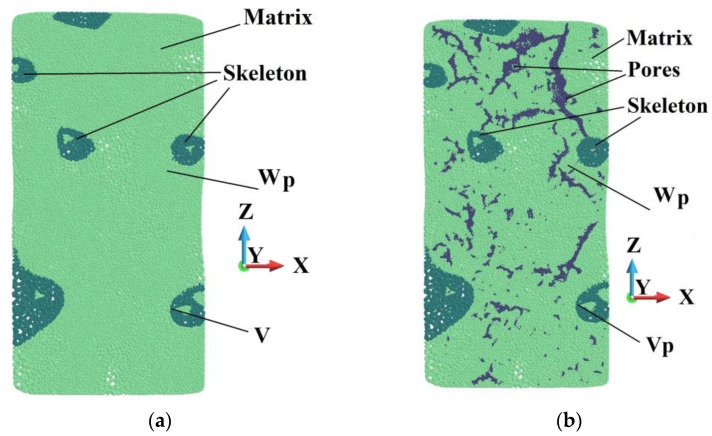
Vertical cross-section in the plane *xz*: (**a**) ideal matrix; (**b**) porous matrix.

The analysis of vertical cross-sections shown in Figure 30 gives insight into the depth of the sample. Points V and Vp are chosen in the spots of the skeleton where the HMH stress is the highest at the end of the process. They have the same positions. Points W and Wp are in the place where the equivalent plastic strain is the highest in the porous sample. The spot is close to a void, and Point W has the same position in the sample as the ideal matrix.

**Figure 31:**
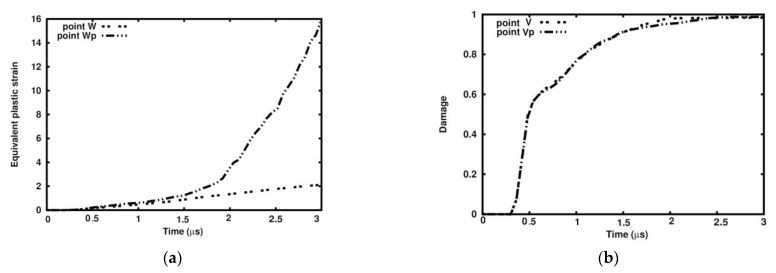
Variable comparison in ideal and porous samples at Points U, Wp, and V, Vp: (**a**) equivalent plastic strain; (**b**) damage parameter.

Figure 31a presents the equivalent plastic strain development at Points W and Wp, which have a high plastic strain concentration. The equivalent plastic strain rapidly grows starting from 1.5 µs up to 15.0 in the porous sample, while in the ideal sample, it grows up to 2.0, giving the ratio 7.5. The damage parameter at Points V and Vp grows to 1.0 at the end of the time interval along practically the same path with minor deviation close to 2.0 µs (Figure 31b).

**Figure 32:**
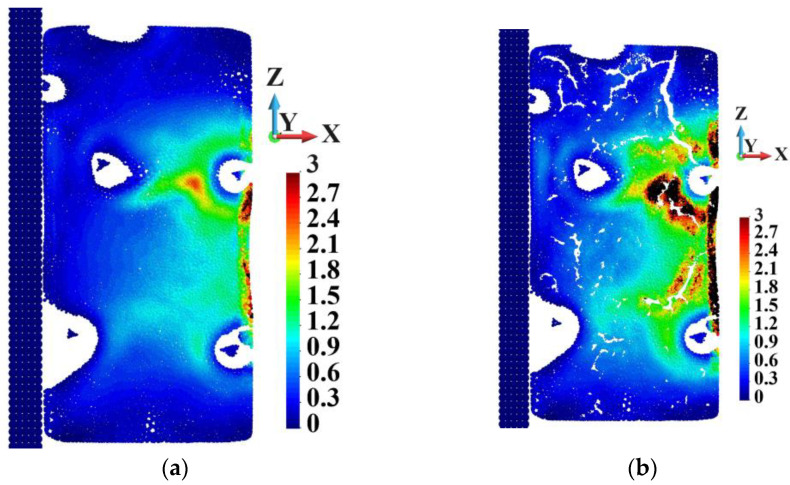
Equivalent plastic strain distribution in vertical cross-section (matrix only), undeformed configuration: (**a**) ideal; (**b**) porous.

Further, Figure 32 compares the distribution of the equivalent plastic strain in the vertical cross-section in an undeformed configuration. It is noted that the plastic strain is concentrated close to the surface of the samples and around the pores in the imperfect sample. The region where the plastic strain is higher than 3.0 is much more significant in the porous sample than in the ideal one.

**Figure 33:**
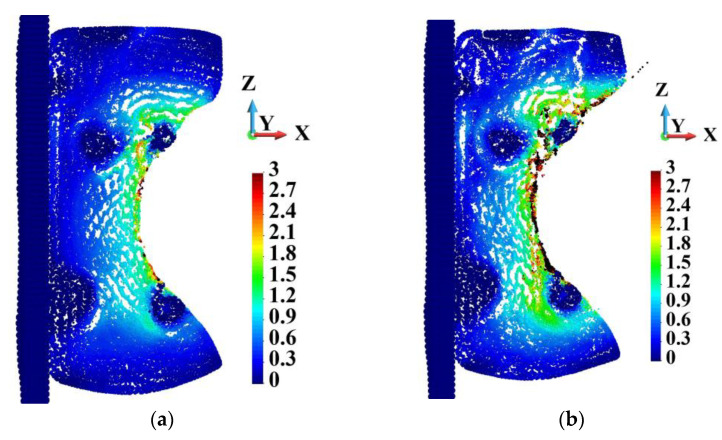
Equivalent plastic strain distribution in vertical cross-section, undeformed configuration: (**a**) ideal; (**b**) porous.

Finally, the deformed configuration of the cross-section is shown in Figure 33. The shapes of the ideal and porous cross-sections are different. Moreover, fragmentation is visible in the porous sample. The area where the equivalent plastic strain is higher than 3.0 is more significant in the porous sample than in the ideal one. The high equivalent plastic strain in the sample with the ideal matrix is concentrated very closely to the surface of the deformed cross-section. The picture of the porous sample is different. Namely, the high equivalent plastic strain distinctly reaches the sample’s interior, which is also compatible with Figure 32b. The white areas that appear horizontally close to the base in both samples are more significant in the porous sample. This shows a tendency to spallation.

### 4.5. Study Limitations

The study is directed to evaluate porosity’s effect on the composite sample’s behavior under impact conditions. Therefore, several simplifying assumptions have been imposed to limit the number of parameters affecting the process. The simplifications concern the materials and the modeling. Namely, the impactor and the base are considered ideally elastic. The skeleton is without pores. There is no transition zone between the phases. The simplifications enhanced the porosity effect, showing the quantitative and qualitative differences between the ideal and the porous samples.

### 4.6. Further Directions

The research will aim to fill the gaps pointed out in Section 4.5. Further research will focus on evaluating imperfections’ influence on the load-carrying capacity of the IPCs. In particular, the porosity of the skeleton will be considered. Then, a viscous-elastic-plastic model of the matrix with shear banding will be introduced [82,83], and the interphase properties will be considered [57,84,85]. The last predicted development step will be introducing the inelastic properties of the impactor and the base.

## 5. Conclusions

To our knowledge, only a few authors were interested in three-dimensional computations of voided media under dynamic loading carried out by a finite element analysis. For example, the impact velocity creates inside material plastic shock velocity, which depends on material parameters and the initial porosity (cylindrical voids) [86,87]. The experimental and numerical models of defective rocks (initially fractured, porous, and with faults) were investigated in [88,89,90]. A static analysis of IPC structures based on a variety of periodic cells was examined in [43,91]. The hypervelocity impact testing of open porosity cast AlSi cellular structures was investigated in [92]. The ballistic resistance of aluminum foam in the sandwich panel considering porosity was experimentally and numerically analyzed in [93]. The ballistic limit velocity strongly depends on the density and thickness of the foam core. Contrary to the above analyses, the description of the IPC’s behavior under impact loading is very limited.

Therefore, the paper’s primary goal is to present the effect of the matrix’s initial porosity in an interpenetrated composite. The internal structure of the sample is obtained by employing computational tomography, which can also be used to obtain the distribution of porosity in the matrix of realistic samples. Then, an image of the samples’ behavior is obtained by employing numerical simulations. A comparison of the behavior of the samples with the ideal and porous matrices is presented.

In the brittle skeleton, the damage parameter and Huber–Mises–Hencky stress are observed. The equivalent plastic strain is the main parameter characterizing the response of the elastic–plastic matrix. The shape of the sample is also observed.

It is found that the matrix’s porosity does not strongly affect the skeleton’s behavior. The matrix’s porosity does not influence the damage parameter distribution and its variability in time in the selected points. This also concerns the Huber–Mises–Hencky stress.

The matrix’s porosity significantly affects its behavior. The maximum equivalent plastic strain is higher in the porous sample than in the ideal one. The distribution of the equivalent plastic strain is smoother in the ideal sample than in the porous sample. The equivalent plastic strain is concentrated close to the pores in the porous sample.

The shape of the ideal and porous samples at the end of the observed time interval also differs. The displacements of the porous sample are more significant than the ideal one. The porous sample tends to be fragmented. At the bottom of the sample, the tendency to spallation is also visible.

## Data Availability

The data presented in this study are available on request from the corresponding author. The data is not publicly available due to ongoing project.
